# Intestinal occlusion by stenotic neuroendocrine tumours of left colon and concomitant association with small bowel gastrointestinal stromal tumours: A case report

**DOI:** 10.1016/j.ijscr.2018.10.034

**Published:** 2018-10-26

**Authors:** M. Amoruso, V. Papagni, A. Picciariello, V.L. Pinto, D. D’Abbicco, A. Margari

**Affiliations:** Dept of Emergency and Organ Transplantation, General Surgery Unit “G. Marinaccio”, University “Aldo Moro” of Bari, Bari, Italy

**Keywords:** NET, GIST, Colorectal, Adenocarcinoma

## Abstract

**Introduction:**

The association between Gastrointestinal Stromal Tumours (GIST) and Neuroendocrine Tumours (NET) is very rare. These tumours have various clinical expressions and sometimes are asymptomatic. Synchronous NETs and GISTs have been already described in literature in few case reports. On the other hand, there is no mention of concomitant presence of discending colon NEC-G3 and small intestinal GIST.

Presentation of case: we presented a case of a patient with clinical evidence of intestinal occlusion and radiological and intraoperative aspects of an adenocarcinoma of the left colon with a single metastasis on small bowel. The pathology analysis of the tumour showed a stenotic left colon NEC-G3 and a small bowel GIST.

**Discussion:**

In this case report GIST was surgically treated as a small bowel ripetitive lesion and NET as a left colon adenocarcinoma. These tumours may have a similar presentation in terms of symptoms, endoscopic findings and imaging results.

**Conclusion:**

Concomitant NEC and GIST is rare but it is important to investigate patients before surgery in order to distinguish these from other tumours because of the different prognosis.

## Highlights

• Gastrointestinal stromal tumours (GIST) and Neuroendocrine tumours (NET) of the gastroenteropancreatic tract are rare neoplasms.
• Treatment for colonic NETs was similar to colonic adenocarcinoma, with segmental resection and lymphadenectomy.
• The associationof GIST and NET is difficult to suspect Serum CgA remains the most important biochemical marker in the diagnostics, monitoring, and establishing the prognosis in colorectal NETs.
• Early diagnosis of concomitant tumors such as GIST and NET will guarantee a better outcome of patients.

## 1 Introduction

Gastrointestinal stromal tumours (GIST) and Neuroendocrine tumours (NET) of gastroenteropancreatic tract are rare neoplasms, showing respectively an incidence of 1% [1] and 2% [2]. Their clinical symptoms are various, depending on stage and primary site.

30–40% of GISTs develop in small bowel [3] and, usually, they have a late clinical expression (between 5^th^ an 7^th^ decade).

Neuroendocrine tumors (NETs) are more common in the small bowel, while colorectal neuroendocrine tumors CR-NET are heterogeneous and infrequent. Colonic NETs close to the rectum are rarer and tend to be more aggressive [4].

Their radiological identification is difficult because there are no specific signs and, most of times, only the entire bowel palpation allows to identify them [5].

We report a case of a 62-year-old with concomitant stenotic left colon NET and small bowel GIST. This case is written according to SCARE criteria [6].

## 2 Presentation of case

A 62 years old caucasian female presented to our Operative Unit complaining of severe abdominal pain in the previous 24 h and of faecaloid vomiting. The patient maintained that she did not have any flatus or bowel movements in the previous 12 h. Furthermore, she referred to have nausea and diarrhea alternated with constipation in the last year, reason why she was waiting for a colonoscopy in the following days.

No previous surgery was reported in her medical history; she only had hypertension treated by beta-blockers and calcium channel blockers.

Patient’s vital signs were: BP 162/100 mmHg, PF 74bpm, temperature 367 °C, RR 20, SpO2 97%, weight 64 Kg and height 166 cm.

Her physical examination showed a tenderness of the abdomen, with no evidence of peritonitis, absence of bowel sounds and rectal exploration was negative.

At the First Aid an ECG and an abdomen X-Ray were performed. The radiograms showed a widely dilated bowel with multiple air fluid levels.

**Fig. 1:**
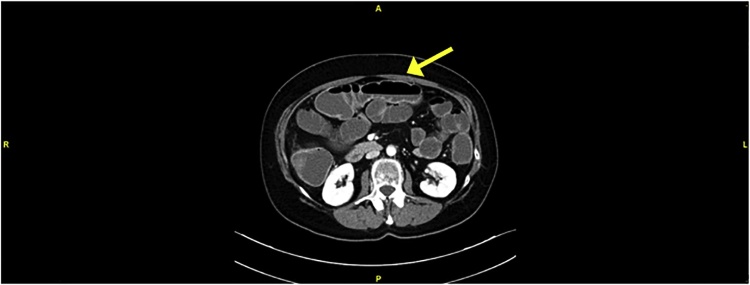
Dilation of small bowel.

**Fig. 2:**
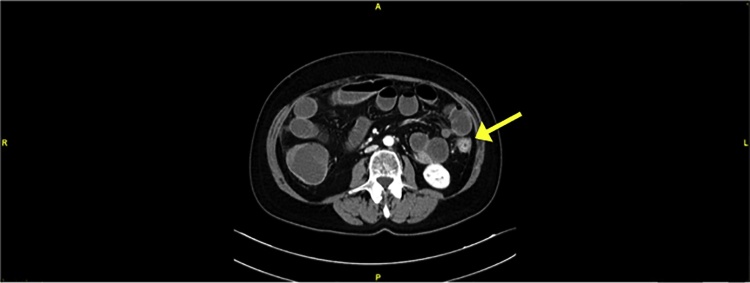
Concentric thickening of the first third of the descending colon.

Then the patient underwent an abdomen and pelvis CT, that confirmed the dilation of the small bowel and revealed a concentric thickening (heteroplastic nature) of the walls of the first third of the descending colon. The stenosis extended for about 3,3 cm with an associated inhomogeneity of perilesional tissue (Fig. 1, Fig. 2).

Blood tests were performed with evidence of hyponatraemia (131 mmol/L) and hypocalcaemia (ionised-Ca: 4.05 mg/dL) with renal impairment (S-Creatinine: 0.91 mg/dL; eGFR: 68 mL/min; S-Urea: 67 mg/dL), neutrophilic leukocytosis and a modest increase of C-reactive proteine (10.7 mg/L). Oncological markers at the entrance were completely negative (CEA: <0.5 ng / mL, AFP: 2.1 ng / mL, CA-125: 20.5 U / ml, CA-19.9: 6.2 U / mL). ASA score of patient was III.

During the exploration of the abdomen, the patient had a distension of the ileal and colic loops above the stenotic left colon tract.

Furthermore, an extraluminal nodule (2.3 cm), maybe a ripetitive lesion, was found on jejunal loop. It was not reported on CT. A small bowel resection of this region was performed and then a Hartmann procedure was carried out. The operation lasted 190 min.

Post operatively, the patient had a protract hypotensive state (mean values 90/60 mmHg).

There were no complications of surgical interest. On postoperative (POD) day 2, the patient presented a temperature of 382 °C and she was treated by antipyretics. An urine culture test was performed with evidence of Enterococcus faecalis. Then a targeted antibiotic therapy was administered.

Diet was advanced as tolerated and patient was discharged on POD 17 after achieving an adequate nutritional status and without any antihypertensive therapy.

The pathology analysis revealed a poorly differentiated neuroendocrine adenocarcinoma (NEC-G3) of the discending colon with endovascular invasion and full-thickness infiltration of the muscular wall, involving the pericolonic adipose tissue with 13 metastatic lymph nodes (pT3N2b).

The jejunal nodule, suspected of repetitive lesion, was a low-grade recurrence GIST.

**Fig. 3:**
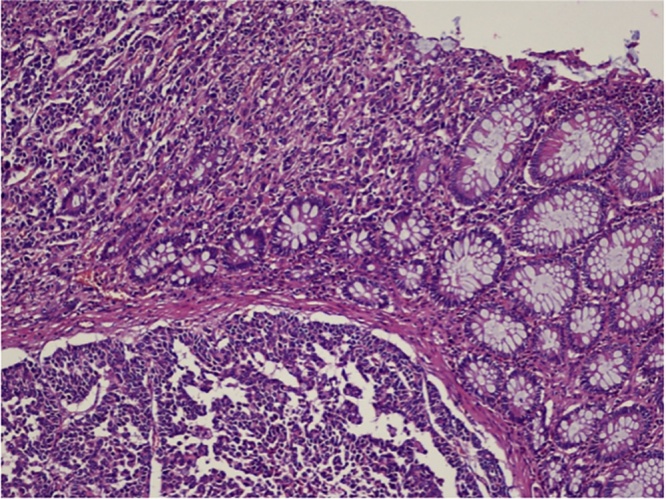
Immunophenotypical aspect (macro).

**Fig. 4:**
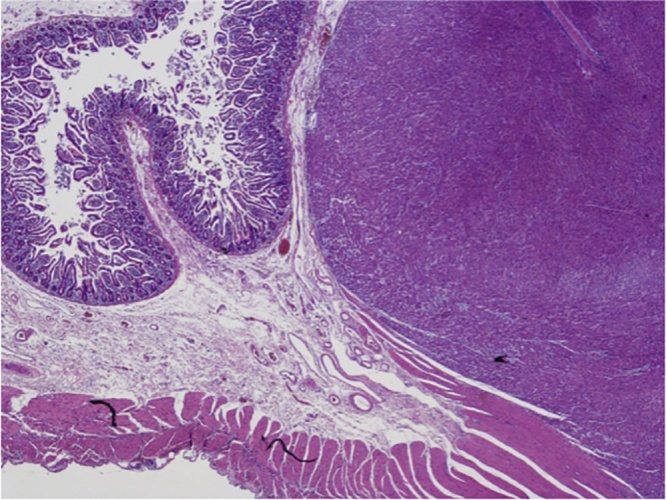
Immunophenotypical aspect (micro).

The immunophenotype analysis showed that NEC was CK20 +, Sinaptophysin +, and focally positive for CD56 (negative for CK7 and Chromogranine) with an Ki67 of 80%. GIST was positive for CD117, CD34 and negative for Smooth Muscle Actin (SMA) with mitotic counts of <5/50 hpf. (Fig. 3, Fig. 4)

At the discharge, Neuron Specific Enolase (NSE) and Chromogranin A were dosed. They resulted negatives (CgA: 1.6 nmol / L; NSE: 13.1 ug / L).

On POD 22, the wound healing process was normal without local complications. Furthermore, the patient reported a normalization of blood pressure values and started chemotherapy.

## 3 Discussion

The coexistence between GISTs and other neoplasms is pretty rare, ranging from 4.5 to 33% of cases [7].

Gastrointestinal stromal tumours (GISTs) are infrequent, generally solitary lesions with an incidence of around 1/100 000 per year. The most common site of this tumour is stomach (60%), followed by small intestine (30%), colon–rectum (5%), and esophagus (1%). A small percentage of GISTs has been described in the omentum and mesentery (4%) [8].

Usually their surgical treatment consists of simple enucleation or segmental resection.

Neuroendocrine gastroenteropancreatic tumours (GEP-NETs) are a heterogeneous group of tumours with their origin in neuroendocrine cells of the embryological gut. Most commonly, the primary lesion is located in the gastric mucosa, the small intestine or pancreas. Colorectal location is uncommon but its rate of detection is increasing thanks to the widespread use of colonoscopy.

Rectal neuroendocrine tumors are more frequent (137% of NET), usually small lesions and their histological malignancy is low to moderate (G1, G2), whereas colon NET are rarer (7,8%), aggressive, poorly differentiated and more malignant (G3) [9].

Colorectal NETs have non-specific symptoms such as obstruction, hematochezia, pain, weight loss and changes in bowel habits [10].

Given that most of colonic NETs are right sided, they may grow considerably before symptoms appear. Rectal and left colon NETs may cause constipation, bleeding (rectal NET), tenesmus and, sometimes, pain [11].

Carcinoid syndrome, consisting of flushing, diarrhea, nausea, and cardiac symptoms is very uncommon. Less than 5% of patients with colorectal NETs develops these symptoms [12].

Histological type of neoplasm according to WHO classification, histological grading and immunohistochemical tests (synaptophysin, chromogranin A and Ki-67 proliferative activity, NSE) [13] are important to estimate the prognosis and to choose the best treatment.

Treatment of colonic NETs is similar to colonic adenocarcinoma, with segmental resection and lymphadenectomy [14].

Synchronous NETs and GISTs have already been described in literature in few case reports [15]. On the other hand, there is no mention of concomitant presence of discending colon NEC-G3 and small intestinal GIST.

In this case the patient had a stenotic left colon tumour (reported on CT), but during the exploration of the abdomen a nodular extraluminal lesion was found on an intestinal loop. The surgical approach, considering the patient’s health state and the suspected ripetitive lesion, was the Hartmann porcedure and a segmental resection of the small intestine, in order to allow a rapid start of an adjuvant treatment after surgery.

In fact, the colostomy began to work on POD 2 and the patient underwent a FOLFOX regimen of chemotherapy 20 days after the discharge.

## 4 Conclusion

Since the concomitant presence of GIST and NET is rare, it is hard to suspect their association in a case like the one we presented. However, its surgical approach does not change. In fact, in this case GIST was treated as a small bowel ripetitive lesion and NET as a left colon adenocarcinoma.

Although they may have an expression similar to the more common metastatic adenocarcinoma in terms of symptoms, endoscopic findings and imaging results, it is important to distinguish them because of their different prognosis.

Colonoscopy is always suggested when there is a change in the bowel habitus, since NET can be endoscopically treated when small [16,17]. Even dosage of chromogranin A and NSE at the entrance of patient helps in the NETs diagnosis. In fact, serum CgA remains the most significative biochemical marker in the diagnosis, monitoring and prognosis of colorectal NETs [18].

Early diagnosis of concomitant tumors such as GIST and NET can guarantee a better prognosis of patients.

## Conflicts of interest

No conflicts of interest to declaire.

## Funding source

No funding received.

## Ethical approval

All procedures performed in studies involving human participants were in accordance with the ethical standards of the institutional and/or national research committee and with the 1964 Helsinki declaration and its later amendments or comparable ethical standards.

## Consent

Written informed consent was obtained from the patient for publication of this case report and accompanying images. A copy of the written consent is available for review by the Editor-in-Chief of this journal on request.

## Author contribution

Amoruso Michele: first surgeon, conception of study design, data collection, analysis, manuscript writing, revision and manuscript submission.

Papagni Vincenzo: conception of study design, data collection, analysis, manuscript writing and revision.

Picciariello Arcangelo: conception of study design, data collection, analysis, manuscript writing and revision.

Pinto Vito Leonardo: critical revision of the manuscript, approved the final version of the manuscript for submission.

D’Abbicco Dario: conception of study design, data collection, analysis, manuscript writing and revision.

Margari Antonio: second surgeon, drafting, revising of the manuscript and participation in the care of the patient.

## Registration of research studies

N/A.

## Guarantor

Michele Amoruso MD.

## Provenance and peer review

Not commissioned, externally peer reviewed.

## References

[1] Lai E.C., Lau S.H., Lau W.Y. (2012). Current management of gastrointestinal stromal tumors-a comprehensive review. Int. J. Surg..

[2] Norlen O. (2017). Preoperative (68)Ga-DOTA-somatostatin analog-PET/CT hybrid imaging increases detection rate of intra-abdominal small intestinal neuroendocrine tumor lesions. World J. Surg..

[3] Beham A.W. (2012). Gastrointestinal stromal tumors. Int. J. Colorectal Dis..

[4] Anthony L.B. (2010). The NANETS consensus guidelines for the diagnosis and management of gastrointestinal neuroendocrine tumors (nets): well-differentiated nets of the distal colon and rectum. Pancreas.

[5] Niederle M.B. (2010). Gastroenteropancreatic neuroendocrine tumours: the current incidence and staging based on the WHO and European Neuroendocrine Tumour Society classification: an analysis based on prospectively collected parameters. Endocr. Relat. Cancer.

[6] Agha R.A. (2016). The SCARE statement: consensus-based surgical case report guidelines. Int. J. Surg..

[7] Tavares A.B. (2012). Gastric GIST with synchronous neuroendocrine tumour of the pancreas in a patient without neurofibromatosis type 1. BMJ Case Rep..

[8] Nishida T. (2016). Diagnostic and treatment strategy for small gastrointestinal stromal tumors. Cancer.

[9] Modlin I.M., Lye K.D., Kidd M. (2003). A 5-decade analysis of 13,715 carcinoid tumors. Cancer.

[10] Murray S.E. (2013). Clinicopathologic characteristics of colonic carcinoid tumors. J. Surg. Res..

[11] Yoon S.N. (2010). Clinicopathological characteristics of rectal carcinoids. Int. J. Colorectal Dis..

[12] Modlin I.M. (2005). Current status of gastrointestinal carcinoids. Gastroenterology.

[13] Scoazec J.Y., Couvelard A., Reseau T. (2017). Classification of pancreatic neuroendocrine tumours: changes made in the 2017 WHO classification of tumours of endocrine organs and perspectives for the future. Ann. Pathol..

[14] Plockinger U. (2004). Guidelines for the diagnosis and treatment of neuroendocrine gastrointestinal tumours. A consensus statement on behalf of the European Neuroendocrine Tumour Society (ENETS). Neuroendocrinology.

[15] Ding J. (2014). Synchronous poorly-differentiated neuroendocrine carcinoma and gastrointestinal stromal tumor of the stomach: a case report with immunohistochemical and molecular genetic analyses of KIT and PDGFRA. Int. J. Clin. Exp. Pathol..

[16] Zhou P.H. (2010). Advantages of endoscopic submucosal dissection with needle-knife over endoscopic mucosal resection for small rectal carcinoid tumors: a retrospective study. Surg. Endosc..

[17] Lee E.J. (2013). Endoscopic submucosal dissection for colorectal tumors--1,000 colorectal ESD cases: one specialized institute's experiences. Surg. Endosc..

[18] Kolby L. (2004). Chromogranin A as a determinant of midgut carcinoid tumour volume. Regul. Pept..

